# 0897. Mechanisms of pulmonary inflation during lung injury assessed by synchrotron radiation computed tomography

**DOI:** 10.1186/2197-425X-2-S1-O23

**Published:** 2014-09-26

**Authors:** G Perchiazzi, JB Borges, G Hedenstierna, L Porra, L Broche, M Pellegrini, A Sindaco, AP Tannoia, S Derosa, FF Todisco, T Fiore, A Larsson, S Bayat

**Affiliations:** Bari University, Emergency and Organ Transplant, Bari, Italy; Uppsala University, Dept of Medical and Surgical Sciences, Uppsala, Sweden; University of Helsinki, Dept of Physics, Helsinki, Finland; European Synchrotron Radiation Facility, Grenoble, France; Université de Picardie Jules Verne, Amiens, France

## Introduction

The process of lung inflation during mechanical ventilation is characterized by phenomena of alveolar recruitment and distension but regional interaction and temporal sequence are not known for the core areas of the lung. The only information available are referred to subpleural alveoli, studied by microscopy. Relevance of this issue derives from the notion that 1) radiologic studies reveal that air spaces behavior in the core regions of the parenchyma is very complex 2) unsuited patterns of mechanical ventilation (MV) can trigger a Ventilator-Induced Lung Injury (VILI). Synchrotron Radiation Computed Tomography (SRCT) yields tomographic images at resolutions higher than conventional CT.

## Objectives

The aim of this study was to evaluate the behavior of air spaces using SRCT in an animal model of VILI at different Positive End Expiratory Pressure (PEEP) levels.

## Methods

Six anesthetized, tracheostomized rabbits were mechanically ventilated to preserve healthy conditions (HC) of the lung (Tidal Volume=7ml/kg, PEEP=3 cmH_2_O) and a sequence of SRCT exposures was performed at 40 different consecutive gravitational levels, at PEEP levels of 12, 9, 6, 3, 0 cmH_2_O, during end-expiratory pauses. Then VILI was induced combining repeated lung lavages followed by injurious MV in Pressure Control mode with Peak Pressure=35 cmH_2_0 and ZEEP, for one hour; then the same sequence of SRCT exposures was performed. SRCT images (resolution = 45.5 µm) were transformed into binary images. From each animal, from a stack of 40 images at different gravitational levels, a Region Of Interest (ROI) of 100x200 voxels was studied by using the Image Processing Toolbox for Matlab. The increase of air spaces numerosity (NA) was considered as an index of alveolar recruitment while the increase of air spaces area (SA) and perimeter (PE) as indices of alveolar expansion. The statistical analysis was performed applying the Student's T test (a=0.05) on NA, SA and PE during HC and VILI at different PEEP levels.

## Results

During healthy conditions (HC), SA and PE increased in direct proportion to PEEP up to 9 cmH_2_O, then remained stable; NA increased up to PEEP=6 cmH_2_O and then decreased with higher PEEP values. During VILI, SA, PE and NA increased with PEEP in a uniform manner.

## Conclusions

In HC the changes of shape and size of air spaces seemed to provide a greater contribution to the increase of lung volume than recruitment mechanisms. NA tendency to decrease after a peak was interpreted as a consequence of progressive stretching of interstitial septa with creation of false connections between adjacent air spaces (false derecruitment). In VILI, alveolar recruitment during lung inflation was a continuous pattern throughout the range of PEEP levels that were studied.

